# Effort-Optimized Intervention Model: Framework for Building and Analyzing Digital Interventions That Require Minimal Effort for Health-Related Gains

**DOI:** 10.2196/24905

**Published:** 2021-03-12

**Authors:** Amit Baumel, Frederick J Muench

**Affiliations:** 1 Department of Community Mental Health University of Haifa Haifa Israel; 2 Partnership to End Addiction New York, NY United States

**Keywords:** behavior change, digital health, mental health, addiction, intervention, behavioral health, effort, salience, persuasive design

## Abstract

The majority of digital health interventions lean on the promise of bringing health and self-care into people’s homes and hands. However, these interventions are delivered while people are in their triggering environments, which places competing demands on their attention. Individuals struggling to change or learn a new behavior have to work hard to achieve even a minor change because of the automatic forces propelling them back to their habitual behaviors. We posit that effort and burden should be explored at the outset and throughout the digital intervention development process as a core therapeutic mechanism, beyond the context of design or user experience testing. In effort-focused conceptualization, it is assumed that, even though goals are rational and people want to achieve them, they are overtaken by competing cognitive, emotional, and environmental processes. We offer the term effort-optimized intervention to describe interventions that focus on user engagement in the face of competing demands. We describe design components based on a 3-step process for planning an effort-optimized intervention: (1) nurturing effortless cognitive and environmental salience to help people keep effort-related goals prominent despite competition; (2) making it as effortless as possible to complete therapeutic activities to avoid ego depletion and self-efficacy reduction; and (3) turning the necessary effortful activities into sustainable assets. We conclude by presenting an example of designing a digital health intervention based on the effort-optimized intervention model.

## Introduction

Effort is physical, mental, or emotional exertion in an attempt to meet a goal. The effort exerted by an individual depends on the interplay between internal (eg, cognitive ability, motivation) and external (eg, social and environmental) facilitators and the barriers between an individual and their desired objective [1,2]. Substantial research has highlighted that reducing the effort needed to achieve an objective will increase the likelihood of achieving that objective in contexts ranging from consumer behavior to friendship [3]. The modern consumer technology industry has essentially been built on the premise of reducing the complexity and number of steps needed to reach desired objectives. Subsequently, the majority of digital health interventions have leaned on the promise of bringing health and self-care into people’s homes and hands, overcoming the barriers to traditional services such as distance to a clinic, transportation, childcare, and more [4-8].

Despite the fact that digital interventions have significantly expanded their reach, with tens of millions of app downloads, the retention rates across the range of digital interventions remain very poor, with only 4% of behavioral health app users continuing use after 15 days [9]. Subsequently, findings suggest that user engagement with a digital intervention is 4 times higher under trial in comparison with the use of the same intervention in the real world [10]. A recent report [11] on the use of MindSpot, an Australian digital mental health service may shed light on this phenomenon—the researchers reported an increase in the proportion of users looking for confidential assessment and a substantial decrease in the proportion of users looking for a traditional course-based internet intervention [11]. This suggests that many users are expecting far shorter therapeutic encounters such as microinterventions [12] compared with what would have been traditionally expected from users engaging digital health interventions.

We propose that a primary challenge with user engagement in digital interventions is that individuals who are struggling to change must work hard to achieve even a minor change because of the automatic forces propelling them back to their habitual behaviors [13-15]. Substantial literature has emphasized the continuum of automatic processes driving psychological distress and effortful processing fostering psychological health [16,17]. Not surprisingly, those with severe addiction and mental health disorders typically require a higher level of care (eg, inpatient care) to reduce the severity of symptoms in a controlled environment where recovery is the most salient cue. In effect, individuals have the headspace to work on their goals without being bombarded by environmental cues [18].

An effort-focused intervention model changes our conceptualization in the sense that we assume that goals are rational and that people want to achieve them. However, competing events in people’s lives either require less effort or are more salient. As the next generation of digital health interventions is developed, we argue that an exploration of effort and burden should form the baseline for intervention development.

## Existing Literature on Effort Reduction

The fields of user experience, heuristic evaluation, and persuasive design focus on principles such as user control, simplicity, predictability, and satisfaction specifically designed to increase engagement [19-21]. The outputs of these efforts range from autofill opportunities to 1-click shopping and frictionless feeds. In behavioral economics, effort reduction is often achieved with a default option [22]. For example, in their seminal paper on organ donation, Johnson and Goldstein [23] posited that one of the mechanisms of increased donations is that “making a decision often involves effort, whereas accepting the default is effortless.” Environmental engineering theory, popularized by books [24], and seminal studies [25] on manipulating availability and access to different foods have revealed that reducing cognitive effort by making healthier choices available and unhealthy choices more burdensome to obtain improves healthy behaviors significantly and unconsciously. Underlying gamification presents perceived effort reduction by enhancing reward and reinforcement while pursuing a goal in a fun and engaging way [26]. Subsequently, a recent review [27] has shown that clinical applications that reduce the effort required from participants to engage in a desired response decrease self-injurious behavior, decrease pica, and increase appropriate eating.

These approaches are used often in the digital behavior change, supplemented by targeted persuasive intervention design for behavior change. For example, the Fogg model introduced the concept of the trigger into social-cognitive theory [28,29], that is, triggers presented at the right time in the right context reduce cognitive effort and increase motivation. More comprehensive taxonomies and persuasive models have been developed to identify core elements of behavior change interventions that drive engagement. For example, Oinas-Kukkonen and Harjumaa [30] developed a set of principles to build sustainable interventions that include concepts such as tunneling and choice reduction to foster engagement. Michie and colleagues [31] have developed a set of core behavior change principles, in which effort-reduction is implied, to guide intervention development heavily focused on learning theory and shaping behavior.

One of the reasons text-messaging interventions are acceptable may not be because they are just-in-time interventions, but rather, because individuals do not have to do anything except passively receive a text message once they sign-up. Text-messaging interventions have higher engagement over time than app-based interventions for perhaps no other reason than their effortlessness. For example, after 10 months of being signed up for the Text4Baby SMS intervention, 74.4% of mothers were still receiving messages [32]. To further increase sustained engagement the study [32] reported that “the extra step required to update the service with the birth date is being removed in case this has been a barrier to maintaining participation.”

We are not positing that the focus on effort reduction is a new phenomenon. We are suggesting that effort reduction is often overlooked by our field as we develop interventions from the outset and at every stage of intervention engagement. The theories described above such as tunneling, persuasive design, and gamification are methods that reduce effort as a passive result of the optimal state rather than by the design goal of fostering such a state. If we focus on gamification alone, for example, we may miss opportunities for effort reduction at every stage of the behavior change process; however, if we focus on effort reduction, gamification will likely be included as one task within a larger effort-optimized intervention model.

## Effort Optimized Intervention Model: Fostering Effortful Behavior by Making it as Effortless as Possible

**Figure 1:**
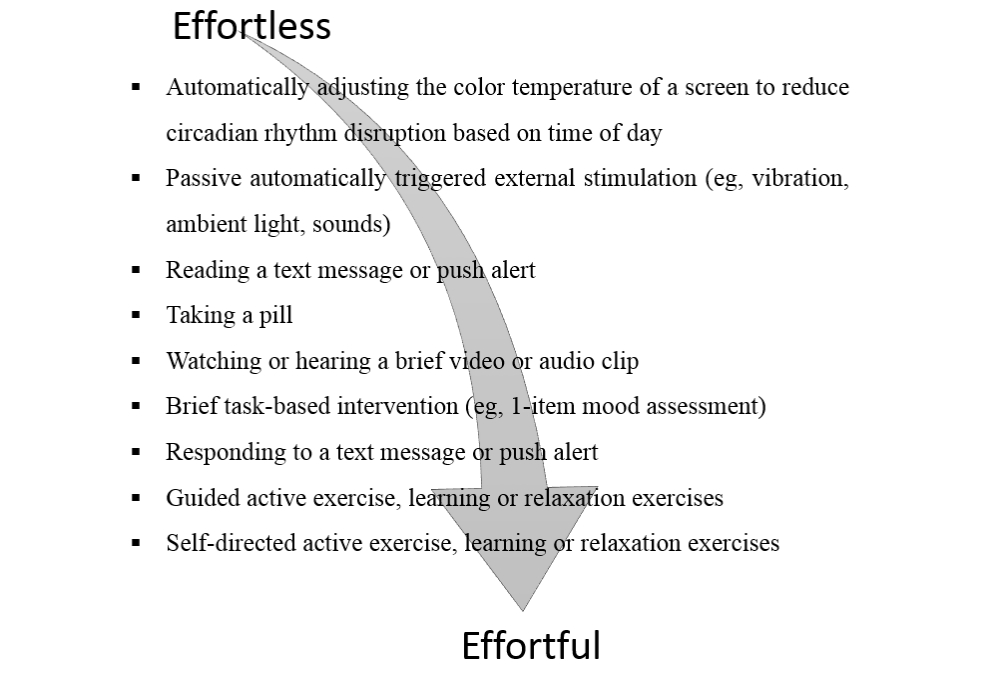
Effortless to effortful intervention examples.

We offer the term *effort-optimized intervention* to describe interventions that focus on generating engagement with processes of therapeutic change in the face of competing demands. Here *engagement* refers to the time window of the intervention itself which may vary—mostly stretching from days to months [12]—and refers to the notion that the user has to engage with the intervention for the targeted time window for it to reach a desired impact. Understanding effort optimization starts with the question “what is the lowest burden method to trigger behavior change?” For example, if one is trying to reduce arousal before bedtime, interventions may require a range of engagement levels (Figure 1).

Exploring the continuum of required effort enables the selection of interventions that meet individuals where they are in terms of motivation, ability, and barriers [28]. A person with almost no self-efficacy in changing a behavior may easily change the display options on their mobile phone to reduce blue light after 8 PM but may be unlikely to engage in guided paced breathing. At the same time, there may be no barriers to creating additive models of effortful engagement for those who are motivated and engaged. Unfortunately, the majority of effort targets for behavioral and mental health have fallen on the higher end of the spectrum. As a result, we are required to optimize effortful behaviors in times when we cannot make them fully effortless or passive.

We describe a 3-step process in the design of an effort-optimized intervention sequence, involving (1) nurturing salience to increase the chance of desired behaviors occurring in the face of competition; (2) making it as effortless as possible to complete therapeutic activities in order to avoid ego depletion and self-efficacy reductions; and (3) turning the necessary effortful activities into sustainable assets.

## Nurturing Salience to Increase the Chance of Desired Behaviors Occurring in the Face of Competition

### Background: What Makes a Therapeutic Target More Salient?

*Salience* refers to how much a certain object, either internal or external, is prominent in one’s mind. Salience can be triggered via a range of experiences—from an intense emotional event that becomes deeply encoded in memory to ongoing subtle cues embedded in one’s daily routines over long periods of time. For the purposes of this paper, we discuss the latter trigger. (We use the term *trigger* to refer to the broad category of digital stimuli designed to prompt desired actions and reactions from users [33].) An object’s salience can be defined based on its availability, whether it is actionable, and how much it is linked to a reward. (Table 1 contains a summary of components described with the body of this manuscript.)

**Table 1:** Summary of the effort-optimized intervention 3-step design process and related components.

Component			Explanation		Example
**Nurturing salience**					
	Increasing task or goal availability	How easy is it to think about the goal compared to competing demands at a desired time point?		Just-in-time text messaging or push notifications about the targeted task; using implementation intentions to mark dinner as an environmental cue for a parent to conduct a family gratitude exercise	
	Creating an actionable script	Triggering a step-by-step script to convey exactly what to do to foster the automaticity of the script during the task		Embedding a simple step-by-step app guidance for parents learning what to do in the face of their child’s panic attack	
	Incentive salience for a task or goal	A cognitive process that includes an automatic motivational component that links a person’s desires to a rewarding stimulus to create a feedback loop toward behavior change		Offering a meaningful immediate reward through the app such as celebration of a successful running exercise	
	Optimizing novelty	Avoiding habituation by not presenting similar stimuli over and over again and varying the affective impact on the individual		Changing the delivery medium of inspirational motivational messages from text, video, and audio across a program	
**Making the completion of therapeutic activities as effortless as possible**					
	Setting graded tasks	Determining small and achievable goals, and moving forward in small steps		A mobile app beginning running distance at 0.5 km and gradually stepping the user up to 5 km	
	Setting dynamically tailored tasks	Adapting to the user’s state based on passive data tasks they care about and past failures and successes		When it takes more time for the user to acquire a skill, they receive additional features from the program prior to moving forward.	
	Reducing the effort required to engage in therapeutic activities	Keeping all relevant tools available in-house; making it as easy as possible to perform the activity		Taking a photo of a meal through the app which analyzes it to document calorie intake; automatically triggering changes to screen color temperature based on time of day	
**Turning effort into assets**					
	Documenting and reflecting on past effort-related activities in a meaningful way	Turning effort into assets by documenting and reflecting on aspects users care about during the therapeutic process; once assets are made, users are inclined to keep investing so that their assets will not go to waste		Presenting effortful activities the user conducted (eg, user reports on socializing with a friend) and how these activities are helpful (eg increase life satisfaction other time)	
	Turning effort into a meaningful narrative	Helping people acknowledge the link between the effort they just exerted and their commitment to the therapeutic process		Upon reporting a positive interaction with their child, parents are asked to celebrate investing effort in becoming better parents	
	Reframing effort as positive	Embedding a narrative in which the reward is the respect for asserting effort beyond skills acquisition		Encouraging users who finished an online learning on coping with depression by stressing out how this activity shows their commitment to feeling better	

Availability is the ease with which one is able to think about the target object at a given time point. A simple way to manipulate an object’s availability is to trigger it using just-in-time mobile reminders or environmental triggers [33]. Critically, availability can be manipulated cognitively by priming people to think in a certain manner at a given time point, thus creating automaticity [34]. Presenting a certain object can prime a goal-directed behavior in the direction of the desired target, whether it be by using words associated with homophobia to increase implicit antigay bias [35], holding a warm object to increase altruism [36], or using the words “substance abuser” or “person with a substance-use disorder” to manipulate individuals’ assumptions about whether someone should go to jail or to treatment [37]. These examples are congruent with the notion that creating cognitive prominence can trigger goal-directed behaviors without having to overtly instruct someone to be more mindful of a goal. Availability can also be triggered overtly through motivational reminders and environmental cues embedded in just-in-time digital interventions [38]. The end goal is to increase goal availability during an effortful decision by reducing the amount of effort needed to retrieve the information.

An object is actionable when the person knows exactly what to do to achieve the desired outcome and how to do it at a given time point. The steps must become salient so that some action can be taken when goal-striving is triggered. Implementation intentions are priming methods that create if–then statements to trigger attention for a future desired outcome by making the association between a trigger and the resulting step-by-step behavior more immediate and less effortful [39,40]. Implementation intentions consist of a basic 2-step process to increase actionable behavior toward a goal, for example, (1) “when I sit down for dinner” (trigger); (2) “I will ask everyone to talk about one thing they are grateful for before I put the first bite in my mouth” (script). Other examples include online graphic illustrations and scenario-based scripts [41] to accompany text guidance. Using graphics targets different memory mechanisms and can help make a script more accessible from multiple pathways.

The rewarding aspect, defined as *incentive salience*, is a cognitive process that includes an automatic motivational component that links a person’s desires or actions to a rewarding stimulus [42]. Incentive salience creates a feedback loop by which promise of the reward drives a person’s attention. When it comes to behavior change in a person’s natural environment, we assume that the reward for maladaptive behaviors such as parents yelling at their kid to “shut up” will be immediate (silence). Changing a person’s behavior to adopt better practices requires significant effort and the promise of long-term rewards (eg, reducing behavior problems). Technological advances such as immersive virtual reality experiences, neurostimulation, and even actively targeting incentive salience by manipulating immediate rewards can create a reward-based feedback loop for behavior change. Immediate rewards can be produced by rewarding the attempt at the behavior and not the outcome (eg, making sure the parents understand that they are being evaluated based on their responses and not based on their child’s behaviors and immediately celebrating their successes in improving their daily practices).

When we teach or ask the user to conduct a new internal (eg, cognitive reframing) or external (eg, exposure) therapeutic process or activity, the quality of our digital message delivery also affects how salient the targeted process will be in the user’s mind. The more immersive, tangible, relatable, and personally tailored the message is, the more salient the targeted process will be. For example, when teaching a user to conduct an exposure paradigm, using automated scenario-based learning with video tutorials and relatable figures will be more immersive cognitively than explanations with texts; a text correspondence through an automated system that asks several questions and then provides personalized feedback and personalized motivational messaging that are meaningful in one’s life will be emotionally more salient than general statements. Furthermore, because stimulus quality plays a significant role in drawing the user’s attention, we must think about how to avoid habituation by not repeatedly presenting similar stimuli [43]. In effect, using novelty, such as changing the delivery medium, message type, and content, is key to maintaining user attention over time.

Finally, because the developers’ goal is to design an effort-related intervention sequence that is sustainable in people’s lives, they have to think about embedding these activities in an environmental context that will then serve as a natural environmental cue [44]. This enables the desired activity to be automatically triggered without having to draw attention to it—a requirement in the first steps of process acquisition. For example, when a desired positive interaction is to share a funny story with their child, parents could be prompted to do this during dinner. In this case, the developer views dinner time as an environmental cue.

From a public health perspective, when an individual is required to engage with a digital health intervention, it also means that unhealthy cognitions become more salient in the individual’s mind—they are available, rewarding, and actionable—otherwise, this person would not need an intervention. Therefore, examining the interaction between salience, effortful behavior, and motivation can help us to understand the type of salience manipulation needed in a particular intervention sequence.

### Promoting Desired Activities

**Figure 2:**
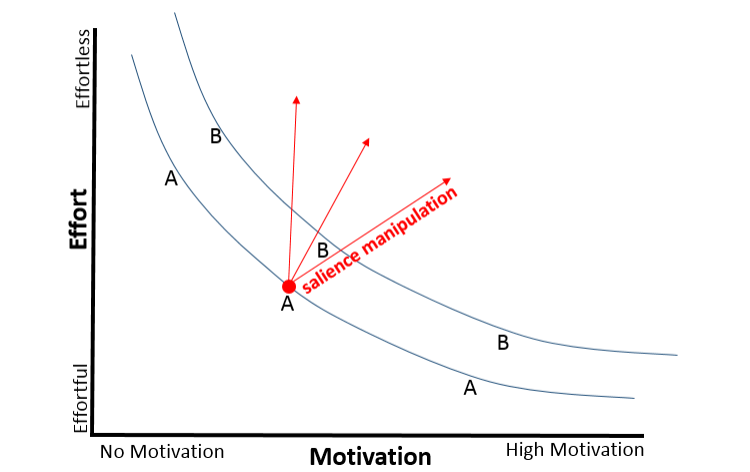
The probability of an activity occurring in the face of competition as a function of effort, motivation, and salience. The blue curves are probability curves.

Figure 2 presents a model that describes the probability of an activity occurring in the face of competing activities as a function of effort, motivation, and salience. This conceptualization follows Fogg’s [28] work on determining the probability of a behavior occurring following a trigger based on the relationship between ability and motivation. We use effort instead of ability to stress the importance of subjective experience, which can fluctuate mainly due to levels of effort expenditure prior to a task and the available ego strength available to complete a task.

As shown in Figure 2, the probability of a behavior occurring is based on the relationship between effort and motivation. Activities located on the same curve have the same probability of occurring either because they are less effortful or because they are more motivating. Furthermore, if 2 prompted events compete over resources (eg, whether parents either yell at their kids or take deep breaths and try to calmly educate them), the activities located on a higher curve have a higher probability of occurring (that is, B will have a higher chance of occurring than A). Salience plays a crucial role in this process. Manipulating the salience of the desired activity (ie, making it more available, rewarding, and actionable) has the potential to increase the chances of the activity occurring in the face of competition by making the activity either more motivating or less effortful.

Availability increases the effortlessness of the targeted behavior because, in one’s subjective experience, there are fewer competing or available activities. For example, having a playlist on the way home from work that includes a 1-minute audioclip that discusses the desired pre-evening activity makes it more available in the person’s mind when they arrive home than other activities.

Actionability increases both effortlessness and motivation. For example, parents are presented with a tangible video that teaches them step-by-step what to do when their child misbehaves (scenario-based learning), then they must confirm their understanding using a worksheet in which they write their own step-by-step process for the exact targeted behavior and print it out, and later that week, when their child acts in a certain way, they can easily identify the event and know exactly what to do. Consequently, they need to exert less effort to identify the trigger and decide on the action.

Incentive salience involves making the reward clear, tangible, and relatable. For example, a parent drives home from work and is prompted to listen to a 1-minute motivational audioclip on the significance of playing together with their child—an audioclip that also directs them to reflect on their time playing with their parent and how meaningful it was. The novelty and emotional activation of this exercise increases the prominence of the reward and the availability of the desired behavior (which, as suggested, also reduces the effort exerted when performing this activity).

## Making the Completion of Therapeutic Activities as Effortless as Possible

### Overview

Developing models that are available, actionable, and rewarding require some effort at the outset of the behavior change process in order to reduce the effort exerted during daily goal-directed behaviors. This approach must also be accompanied by making the tasks themselves as effortless as possible.

### Setting Graded and Dynamically Tailored Therapeutic Activities as Targets

The literature points to the importance of setting graded tasks, determining small and achievable goals, and moving forward in small steps as the user succeeds in prior steps [45]. Breaking a distal goal into achievable subgoals increases self-efficacy [46], which has been shown to be an important factor in determining whether a task will be initiated and successfully performed. From a behavior change standpoint, when using graded tasks, less effort is required for each activity. This increases the user’s ability to engage in the task, thereby increasing the chances that the triggered activity will occur [28,40,44]. Critically, the use of graded tasks also involves improvement of the user’s skills or condition in such a way that the next task becomes less effortful to achieve, as highlighted in shaping paradigms [47]. For example, when an untrained user is triggered by an app to run 5 km for the first time, the amount of effort required to complete this activity may be very high. However, if the user is triggered to run 1 km several times and then 3 km in a graded manner, the amount of effort related to running 5 km might be as little as it was to run 1 km for the first time.

One psychological technique that is not very common in digital interventions (though it could be easily incorporated) is the presentation of an artificial step prior to a subsequent step that otherwise might demand too much effort to complete. For example, when parents are taught to present an appropriate nonharsh consequence in the event of their child’s disobedience [48,49], this step may feel like a giant leap, thus demanding plenty of effort in the parents’ mind. We may, therefore, create another step of setting expectations for which parents are directed to sit with their child prior to changing the way they react to them and to simply present the fact that they are going to do whatever they can to help them, meaning the rules of the house are going to change. This strategy enables parents to acquire self-efficacy in a small manner prior to anything else and could be easily incorporated in an adaptive digital program.

As implied, the adaptive nature of the tasks involves taking the user’s state into account. User level of motivation is a moderating factor in determining the amount of effort the person is able to exert. Motivation is expected to fluctuate during the intervention based on prior successes and failures [50]. Users who encounter difficulties may require a different task or path than those who found the task easy to complete based on the interplay between self-efficacy, motivation, and goal achievement. Monitoring activities and user condition will enable the task to be dynamically tailored such that the effort required at any given moment is adequate. For example, adaptive goal interventions change the goal based on the user’s successes or failures in achieving the goal, such as by increasing the number of weekly drinks allowed in a drinking moderation intervention when goals are not being met or, conversely, by changing to abstinence if moderation is being met with repeated failures. The real-time adaptation of the digital intervention is effort-optimized to meet the user’s goals, motivation, and commitment [51].

### Reducing the Effort Required to Engage in Therapeutic Activities

Developers can help to make therapeutic activities less effortful by reducing the cognitive and environmental effort required to successfully complete each activity [52-54]. Keeping all the relevant tools for completing a targeted activity within a digital component reduces the cognitive and environmental effort needed to search for those tools elsewhere [55]. It will be easier for users to follow a diet if the diet app makes all the information about the diet, the availability of support groups, and tools to document calorie intake available in-house [55].

Another aspect to consider is whether each tool makes it as easy as possible to conduct the desired activity [56,57]. For example, it would be easier for users to document their calorie intake if they could simply take a photograph of the meal and have the caloric results calculated automatically. Similarly, when training a behavior increases performance, a system can help to reduce the effort required for the training by providing the means to rehearse it [30]. Think, for example, of a person who is trying to overcome social phobia through graded tasks and who is now being asked to chat with a person they do not know in a nonjudgmental environment. In such an instance, providing an option of a click-button within the digital platform to connect them with a trained peer [58] would directly reduce the effort required.

## Turning Effort Into Assets

An effort-optimized intervention does not mean that there is no effort on the part of the user. Indeed, effort contributes to sustainable change because the effort people are choosing or willing to make and the way they perceive it substantially impact the therapeutic process, intervention gains, and future effort capacity. Humans think in narratives with players, good and evil, conflicts, and dramatic changes in the plot [59]. Stories create structure because they have an inner rationale that corresponds with the past-present-future tenses, which enables people to predict the future based on the past. Therefore, people are built to create meaning based on their present experiences in a way that rationally fits with their past story and future direction, as captured in their identity, role in the world, and desires [59].

Effort is a very important ingredient in this process because the effort exerted for an activity will be used to create a meaning that mostly fits within the story we tell about ourselves. Experiments on cognitive dissonance theory and placebo effects strengthen this notion by showing that people who are asked to put more effort into an assignment later perceive it as being more meaningful to them [60]; meanwhile, people who pay more find a placebo to be more helpful [61]. Furthermore, several studies have suggested that people prefer to exert effort on a task when they are motivated to enhance their feelings of relatability, ownership, and control over the potential outcome. A notable example involves a US company that produced instant cakes (“just add water”) in the 1940s targeting women maintaining their households. The product did not sell very well until the company removed some ingredients from the mix, such as eggs and milk, which required the baker to do more work during the baking process. It seemed that, with the increased effort, the baker felt greater ownership over the result and more deserving of the compliments for their work [62]. This phenomenon is described by Ariely as the *Ikea effect*—a cognitive bias that leads us to place higher value on things that we help to create [62].

To summarize, effort is a crucial ingredient in the way we create meaning because the amount of effort we invest in an activity impacts the extent to which we build meaningful stories around that activity. Specifically, the more effort we invest in something, the more meaningful we find it and the more committed we are to it. To take a literary example, when the Little Prince tries to explain to the fox what makes his rose different from the thousands of roses that appear to be identical, “It is the time you have wasted for your rose that makes your rose so important,” he asserts [63].

Understanding this dynamic is key, as interventions that focus only on effort reduction and do not help people to feel that they have choice, acknowledge their work, and therefore, create meaning around the effort-based behaviors may fail in helping people to stay on the beneficial pathway when new challenges arise. In digital health interventions, people’s efforts can be translated into assets by helping them to acknowledge the meaning of their work. In this way, we reframe effort as something positive by stressing that the effort exerted shows the user’s commitment to and ownership of the therapeutic process. This shift toward a growth mindset and meaning-based acceptance can be embedded in all our work to increase effortlessness and decrease efficacy reductions based on an outcome mindset.

People’s efforts can be turned into investment by documenting the aspects they care about during the therapeutic process [55,64]. Because users have already invested in the activity and created some assets, they are more inclined to move forward and keep investing in this path so that their assets will not go to waste. We stress that the desired documentation should be connected to aspects that are highly meaningful in people’s lives, mostly within a social context, such as the time they got to spend with loved ones because they successfully executed an intervention’s task.

These three components of nurturing salience, reducing effort to engage in therapeutic activities, and shifting the meaning of effortful behavior to become an asset can be embedded in both new and existing digital interventions. Whether it be by creating a simple visual diagram of the goal of a lesson at the beginning of a module, playing music in the background randomly to keep users engaged while completing a task, or including a narrative of an effortful journey during periods of declining motivation, we can reduce the effort needed for users to achieve positive outcomes. In turn, this will enable users to achieve their goals without taking away from the core therapeutic skill components of many interventions.

## Designing a Digital Health Intervention Sequence Based on the Effort-Optimized Intervention Model

To further clarify the effort-optimized intervention model and how to design a digital intervention sequence accordingly, we provide an example using common intervention content in parent training programs for young children with disruptive behavior disorders [65-67] aimed at increasing the positive interactions between parent and child. For brevity, we only discuss the aspects of effort optimization during skill acquisition time, not other important aspects such as persuasive design or the therapeutic alliance nurtured between the user and the program [52,68-70]. Our baseline is a standard digital parent training intervention in which parents complete a short interactive module about positive parenting practices. Parents are instructed to increase the positive interactions at home and then directed to the next module a week or two later, depending on success.

Planning an effort-optimized intervention begins with defining the task, considering how it might be perceived by the participant, and identifying competing activities or challenges. In our example, the task is to increase positive interactions when the parent and child are in the same surrounding (eg, at home). For parents, it can be difficult to foster positive interactions because it is not always highly enjoyable at first, especially for those who have not naturally exercised such practices before; parents might not have clear ideas about such interactions and how easily they can be incorporated on a daily basis. Furthermore, such interactions are not necessarily linked to a tangible clear reward. We offer a few common competing events which may require less effort than nurturing a positive interaction with the child (although they are not based on empirical studies, it is important to use clear examples here for didactic reasoning)—by letting a child play a mobile app or watch television which requires much less effort, parents may find that playing with their own mobile device requires less effort and is rewarding in the sense of passing the time, and while parents may have some house chores or work they can do later, finishing them early offers a clear reward. Based on these challenges, developers can use the effort-optimized intervention framework to increase the chances of the desired activity being completed in the face of competition. As shown in Table 2, each concept informs the design of the intervention in a way that is directly related to increasing the chances that users will reach their objective despite competition. First, realizing that parents may want to have positive interactions with their child but lack good ideas, we offer these ideas in a concrete way (eg, ideas for what to discuss in the evening). Second, as we believe that parents may find letting their child watch television to be more rewarding than interacting, we have to address this competing activity both directly by helping parents emotionally connect to the difference between the two activities and indirectly by making the desired activity more salient in their mind. Third, we must acknowledge the parents’ effort in order to create assets that help them feel good about the investment they have made. These considerations result in many new features that are not incorporated in a standard online module-based training environment.

**Table 2:** Features increasing parent–child positive interactions based on the effort-optimized intervention framework.

Component		Plan
**Nurturing salience**		
	Increasing the task’s availability	Triggers with relevant content (eg, ideas for what to discuss during dinner) sent in the hour before parents are home from work Priming parents to ask themselves about opportunities for positive interactions in the face of competing events (eg, thinking that their child would prefer to watch television instead and so not trying) through consistent but variable triggers, such as text questions, motivational scripts, and other minimal cues
	Rewarding/incentive salience	Triggers connecting tangible rewards to the desired activities: “Think of your best memories with your parent. You putting some effort into playing with your child is something that will be far more memorable to you and him/her than times when you both watched separate screens.” Directing parents to celebrate their positive interactions with their kids and to report on it using a mobile app Rewarding consistent attempts at behavior over outcomes through the platform (eg, the outcome is engaging in the behavior, not their child’s behavior)
	Creating an actionable script	A tailored list of positive interactions with brief step-by-step instructions based on an online questionnaire parents were asked to complete.
	Optimizing novelty	Sending all triggers above using different delivery mediums (text, audio, and video), timing (time of day, day, special events), and personas (instructor, peers, celebrity testimonial)
	Embedding tasks based on natural environmental cues	Directing parents to find one positive activity to conduct during dinner, such as a gratitude exercise that can be trigged through the mobile device in the right time
**Making the completion of therapeutic activities as effortless as possible**		
	Setting graded tasks^a^	Asking parents to pick their preferred activities from an list of relevant activities, which automatically creates their own table that is then available on the website and as a printed version
	Setting dynamically tailored tasks^a^	Creating a task list based on efficacy and effort. For example, if the parents report very low efficacy or past failures, a first step may be directing parents to sit with their child when the child is watching television and initiating a conversation
**Turning effort into assets**		
	Documenting and reflecting on past effort-related activities in a meaningful way	Documenting reports in an accumulated manner on the home page of the app or website that offers rewards based on the level of engagement (eg, the amount of quality time reported so far). If not engaged, simple motivational statements replace effortful behavior rewards
	Turning effort into a meaningful narrative; reframing effort as positive	Implementing automated feedback, which presents a narrative of them doing whatever they can to be good parents. For example: “the effort you invested today in trying to play with your kid shows how well you are committed to improve your relationship. You should be proud of yourself.”

^a^Reducing the effort required to engage in therapeutic activities is embedded in this component as well.

## Further Considerations, Future Directions, and Conclusions

Research and implementation of the effort-optimized intervention model demand that considerable attention be paid to some specific aspects. From a theoretical perspective, we need to learn more about what prevents people from performing desired behaviors at the individual level, even when they want to achieve them [2,71]. Studying such instances will enable developers to design user-centric products with relevant effort-optimized intervention sequences. Another line of research could focus on how people sustain beneficial behaviors over time, and more precisely, when and how competing events emerge and what people experience at these times. This knowledge will enable us to understand whether new triggers need to be incorporated into a future time window in order to avoid depletion. Finally, we need to learn how to develop effort-optimized intervention sequences so that they are not intrusive and thus eventually diminish people’s desire and tendency to self-manage their situation.

The mechanistic study of effort reduction has been explored more in the consumer social media and commerce sectors in the form of A/B testing paradigms. In such paradigms, small changes to the user experience are repeatedly tested to optimize engagement, as small changes often lead to massive shifts in engagement (eg, “like” button, frictionless feed, page load time). While there are significant differences among these activities that require little effort with little meaningful long-term reward—and potentially significant negative consequences over time—their success highlights that, to create positive change, our attention as interventionists needs to shift to increase the 3% to 6% engagement rate in health applications. This is especially true given that research has revealed very few differences in outcomes between interventions with differing content or behavior change targets [72]. Effort-optimized intervention paradigms are designed to ensure that equal weight is placed on the content of our interventions and on how we engage and sustain individuals using common processes that adapt to meet individual needs, both in terms of what people need and how they consume and integrate it into their lives.
